# Making Sense of Antisense lncRNAs in Hepatocellular Carcinoma

**DOI:** 10.3390/ijms24108886

**Published:** 2023-05-17

**Authors:** Nicola Mosca, Aniello Russo, Nicoletta Potenza

**Affiliations:** Department of Environmental, Biological and Pharmaceutical Sciences and Technologies, University of Campania “Luigi Vanvitelli”, 81100 Caserta, Italy; nicola.mosca@unicampania.it (N.M.); aniello.russo@unicampania.it (A.R.)

**Keywords:** non-coding RNA, antisense lncRNA, HCC, ceRNET

## Abstract

Transcriptome complexity is emerging as an unprecedented and fascinating domain, especially by high-throughput sequencing technologies that have unveiled a plethora of new non-coding RNA biotypes. This review covers antisense long non-coding RNAs, i.e., lncRNAs transcribed from the opposite strand of other known genes, and their role in hepatocellular carcinoma (HCC). Several sense–antisense transcript pairs have been recently annotated, especially from mammalian genomes, and an understanding of their evolutionary sense and functional role for human health and diseases is only beginning. Antisense lncRNAs dysregulation is significantly involved in hepatocarcinogenesis, where they can act as oncogenes or oncosuppressors, thus playing a key role in tumor onset, progression, and chemoradiotherapy response, as deduced from many studies discussed here. Mechanistically, antisense lncRNAs regulate gene expression by exploiting various molecular mechanisms shared with other ncRNA molecules, and exploit special mechanisms on their corresponding sense gene due to sequence complementarity, thus exerting epigenetic, transcriptional, post-transcriptional, and translational controls. The next challenges will be piecing together the complex RNA regulatory networks driven by antisense lncRNAs and, ultimately, assigning them a function in physiological and pathological contexts, in addition to defining prospective novel therapeutic targets and innovative diagnostic tools.

## 1. Introduction

As the result of pervasive transcription of the mammalian genome, non-coding RNAs (ncRNAs) represent the majority of the transcriptome. High-throughput sequencing technologies unveiled an unprecedent and fascinating picture of transcriptome complexity, where less than 2% of transcription encodes proteins, and a plethora of ncRNA biotypes are found, in addition to the well-known ribosomal RNAs [1]. The finely regulated expression pattern of ncRNAs, further restricted to specific cell types in comparison to coding RNAs, their role in various physiological processes, and the finding of their dysregulation and involvement in pathological conditions have led to a drastic change in the view of ncRNA world: ncRNAs are no longer considered “evolutionary junk” or “transcriptional noise”, but a valuable resource for greater eukaryote complexity, with molecular mechanisms only beginning to be understood [2,3].

Based on their length, ncRNA molecules can be broadly classified into small or short ncRNAs (from few to 200nt) and long ncRNAs ((lncRNAs) longer than 200nt, with a size up to several kilobases (up to 100 kb)) [4].

**Figure 1:**
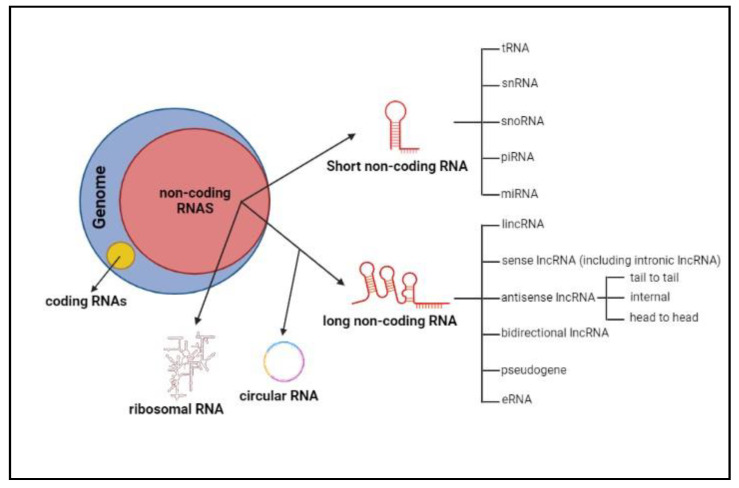
Graphic representation of RNA biotypes’ classification in the transcriptome space. ncRNAs represent the majority of the transcriptome. In addition to the well-known ribosomal RNAs, they can be broadly classified into short and long non-coding RNAs; they can be further functionally categorized as indicated. lncRNAs, the largest class of ncRNAs in the mammalian genome, can be classified into subclasses depending on their genomic locations, origins, and transcription direction. Figures created with BioRender.com.

Short ncRNAs comprise: tRNA (transfer RNA), engaged in translation of mRNA; snRNA (small nuclear RNA), involved in splicing; snoRNA (small nucleolar RNA), involved in ribosomal RNA modification; piRNA (Piwi-interacting RNA), mainly implicated in transposon repression; microRNAs (miRNAs), the most studied group of small ncRNAs, acting as post-transcriptional regulators of gene expression (Figure 1). miRNAs work by driving multiprotein complexes on complementary sequences of target transcripts, thus affecting their translation and/or stability [5]. One miRNA can bind various transcripts, and vice versa one transcript can be targeted by different miRNAs, giving rise to complex regulatory networks controlling more than 30% of protein-coding genes, thus playing key roles in almost all physiological pathways and in the pathogenesis of several diseases [6,7,8].

LncRNAs, the largest class of ncRNAs in the mammalian genome, can be further classified into subclasses, depending on their genomic locations, origins, and transcription directions (Figure 1): long intergenic ncRNAs (lincRNAs) transcribed from intergenic regions that do not overlap any other gene and have their own regulatory elements; sense lncRNAs, transcribed in the same direction as a coding gene, overlapping one or more exons or embedded in one of the introns without touching any exons (intronic lncRNAs); antisense lncRNAs, transcribed as an antisense strand compared to an overlapping known gene; bidirectional (or divergent) lncRNAs, deriving from promoters with bidirectional activity; pseudogenes, a version of coding genes that lost their protein-coding ability due to mutations; eRNAs (enhancer RNAs), arising from enhancers endowed with an enhancer-like function; circRNAs (circular RNAs), deriving from backsplicing events of protein-coding transcripts that form covalently closed continuous loops [2,4,9,10]. LncRNAs are emerging as key gene expression regulators due to their interaction with DNA, other RNA molecules, and proteins, as detailed in the next paragraph.

The specific name of antisense lncRNAs is derived from their sense gene, with the addition of “-AS”; they can be further categorized according to their localization with respect to sense transcripts, i.e., tail to tail (sense and antisense transcripts overlapping in the 3′ region), internal (antisense lncRNA covering the sense transcript), and head to head (sense and antisense transcripts overlapping the 5′ region) [11].

Sense–antisense gene pairs have been found in a significant proportion in the genome/transcriptome of different species, from prokaryotes to mammals (a higher percentage has been found in mammals); furthermore, sense–antisense gene pairs are generally organized into one coding and one non-coding transcript [12,13,14]. Intriguingly, evolutionary conservation of sense–antisense gene pairs has been reported as low, even between closely related mammals; in particular, only 25% of human pairs have both genomic sequence and gene structure conservation in mice [15]. This evidence, along with the poor evolutionary conservation of lncRNAs’ sequence and their role in gene regulation, lead us to speculate that many interspecies differences could rely on the regulation of protein-coding genes, significantly conserved throughout evolution, by non-conserved antisense lncRNAs. In this regard, it has been inferred that human sense–antisense gene loci may be enriched for primate-specific regulatory functions, and antisense lncRNAs could represent the “linchpins of interspecies distinctions” contributing to evolutionary lineage-specific regulatory outcomes and complexity [15].

It is becoming increasingly clear that antisense lncRNAs play key roles, not only in physiological states, but also in pathological conditions, especially cancer. They can contribute to the onset and progression of different type of tumors, acting as oncogenes, oncosuppressors, or both, depending on the type of cancer. lncRNAs have also been implicated in chemotherapy and radiation therapy resistance.

Antisense lncRNAs are also emerging as novel players in hepatocellular carcinoma (HCC); in particular, one study found that they represent 16% of the newly assembled lncRNAs from HCC patients [16]. HCC is one of the most common aggressive human malignancies. HCC ranks third for cancer-related death and is the fifth most common solid tumor worldwide [17]. Viral hepatitis, metabolic syndrome, alcohol abuse, exposure to carcinogenic agents, and genetic disease such as Wilson’s disease and hemochromatosis can cause chronic liver diseases, resulting in more than 80% of human HCCs [18]. In addition to the well-known role of protein-driven processes, functional studies have demonstrated the active involvement of ncRNAs in the regulation of key pathways acting in hepatocarcinogenesis [19,20].

This review aims to explore antisense lncRNA biology, mechanisms of action shared with lncRNAs and unique to antisense lncRNAs, their contribution to HCC hallmarks, and their possible exploitation as innovative biomarkers and potential novel therapeutic targets.

## 2. Biogenesis and Functioning of Antisense lncRNAs

Antisense lncRNAs and, generally, lncRNA biogenesis, share various features with coding RNAs and precursor transcripts of miRNAs: they are transcribed, generally, by RNA polymerase II, subjected to 5′-capping, 3′-polyadenylation, and splicing, since they are mainly composed of two exons [2]. In contrast with mRNAs that move to the cytoplasm for translation, antisense lncRNAs can be retained in the nucleus. Similarly to coding RNAs, but even more distinctly, lncRNAs exhibit highly specific cell lineage and restricted spatiotemporal and tissue type expression patterns, although they are detected in lower amounts [3]. It is still challenging to assign a mechanism/role to the increasing numbers of annotated antisense lncRNAs, due to their lower amounts and poor evolutionary conservation when compared to coding RNAs. In this regard, it should be noted that conservation may be found in secondary structures rather than in sequences; in fact, a crucial feature of lncRNAs is the ability to form thermodynamically stable structures, a structural versatility enabling them to bind to DNA, other RNA molecules, and proteins [21,22]. In addition, an RNA molecule comprising 100nt can capture more than 5 proteins simultaneously, making RNA molecules a more cost-effective scaffold for protein interaction, in comparison to proteins themselves, with well-known modules/motifs dedicated to interactions [23].

**Figure 2:**
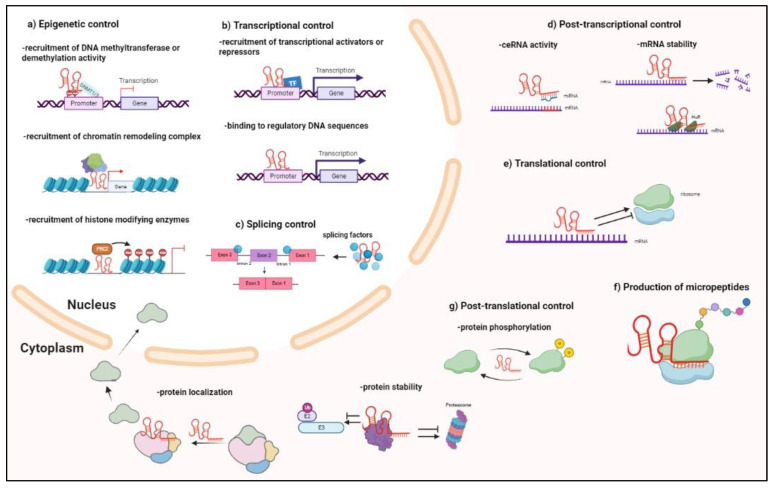
Molecular mechanisms exploited by antisense lncRNAs for gene expression regulation and involved in HCC. Antisense lncRNAs can interact with DNA, RNA, and proteins, thus sharing the mechanisms of gene regulation known for other lncRNA biotypes, and also rerouting them onto their sense genes. Here, the possible mechanisms are represented as compartmentalized between the nucleus and the cytoplasm, with different sublevels (**a**–**g**) to emphasize the idea that the subcellular localization of a newly annotated antisense lncRNA can provide clues about its prevailing mechanism of action. However, a possible shuttling of antisense lncRNA should be considered in diverse physiological and/or pathological contexts. Many of the illustrated mechanisms are involved in the onset and progression of HCC, as detailed in the text. Figures created with BioRender.com.

In the nucleus and cytoplasm, antisense lncRNAs exploit all the mechanisms of gene regulation known for other lncRNA biotypes. However, antisense lncRNAs can also reroute these mechanisms onto their sense genes. In addition, due to sequence complementary, antisense lncRNAs can play a special role regarding sense genes (Figure 2). As a consequence, an efficient manner to obtain clues regarding the main mechanism of action of antisense lncRNAs is to detect their prevailing subcellular localization while taking into consideration a possible shuttling between different compartments under specific physiological or pathological conditions. Then, it is possible to speculate that some of the activities performed in that compartment may involve the regulatory contribution of antisense lncRNAs.

In the nucleus, interaction with DNA, chromatin-modifying complexes, histone-modifying enzymes, and/or various transcriptional regulators determines antisense lncRNAs function as epigenetic and transcriptional regulators (Figure 2a–c). In particular, antisense lncRNAs can recruit DNA methyltransferases to promoters, thus actively contributing to DNA methylation pattern impacting on the expression of a large number of oncogenes or tumor suppressors [24,25]. Antisense lncRNAs can also recruit demethylation enzymatic activities to promoters—as has been demonstrated by RIP (RNA immunoprecipitation) and RNA pull-down experiments—for ZNF667-AS1, able to interact and recruit TET1 to the target gene ZNF667 and E-cadherin to hydrolyze 5′-methylcytosine to 5′-hydroxymerhylcytosine and activate its expression [24,26]. Of note, specific DNA methylation signatures are associated with the HCC stage and patient survival [27,28]. Specific antisense lncRNAs can also interact and recruit chromatin-remodeling complexes and histone-modifying enzymes, such as histone methyltransferase and histone acetyltransferases, that cannot exert their role independently due to a lack of a DNA-binding domain, thus modulating chromatin structure influencing gene expression [11,29,30]. At the transcriptional level, antisense lncRNAs can recruit transcription factors required for promoting or repressing gene expression [31,32]. Although less frequently reported, lncRNAs can also directly bind to genomic DNA to regulate gene expression. An example of this is represented by VIM-AS1 that forms a hybrid DNA:RNA structure, known as R-loop, around the promoter of its head-to-head sense gene VIM, thus triggering an open chromatin structure that favors NF-kB binding and VIM transcription [33]. Another manner of direct lncRNA-DNA interaction is the formation of RNA-DNA triplex, impacting transcriptional induction [34]. Finally, in the nucleus, lncRNAs can also regulate splicing by interacting with splicing factors [20].

In the cytoplasm, by binding proteins and RNA molecules, lncRNAs can regulate gene expression at the post-transcriptional level by sponging miRNAs, regulating mRNA translation and degradation; short open reading frames hidden in the lncRNA sequence could even serve as templates for the synthesis of so-called “micropeptides” up to 100 amino acids long [4,35,36] (Figure 2d–g). Currently, many studies are being published on miRNAs sponging activity of antisense lncRNA and ceRNA activity (competing endogenous RNA): the lncRNA can bind a miRNA and, titrating its availability, can endogenously compete with the other miRNA targets, coding or non-coding RNAs, that are resultingly upregulated [37,38]. In this scenario, all RNA biotypes can modulate each other and design regulatory networks (ceRNET, competing endogenous RNA network) governing different pathways, and whose unbalancing can drive carcinogenesis [39,40]. Even at the post-translational level, antisense lncRNAs can exert control of gene expression by binding and modulating the stability of specific proteins, e.g., protecting from or prompting their ubiquitin-proteosome degradation, and modifying their phosphorylation status, or controlling their localization [11,41].

Finally, sequence complementary allows antisense lncRNAs to have a specific effect on their sense gene. In fact, at the post-transcriptional level, antisense lncRNAs can bind their sense transcript, generating an RNA duplex and affecting the stability of the sense transcript via an RNA interference mechanism; probably the best-known example of this is represented by the pair XIST and TSIX (XIST spelled in reverse order) involved in the X chromosome inactivation, but other examples have been subsequently reported [42,43,44] (Figure 2d). An opposite effect to RNAi can also be observed, due to the stabilization of cognate RNA by interaction with antisense lncRNA [37,38,45].

The above mechanisms have been distilled from many studies, detailed in the next section as related to HCC.

## 3. Antisense lncRNAs Involved in HCC

Antisense lncRNAs are increasingly recognized as mediators of human cancers [11] and, depending on the context, they can act as either oncogenes or tumor suppressors. In the liver, a number of antisense lncRNAs are described as deregulated, thus playing a crucial role in the onset and progression of HCC [16]. In particular, by searching “(antisense lncRNA) AND (HCC)” throughout PubMed, more than 200 articles were retrieved; they were then analyzed and articles concerning antisense lncRNAs were grouped as detailed below. This analysis was also based on information retrieved from https://lncipedia.org (accessed on 3 April 2023) [46].

Here, we first discuss the role and molecular mechanisms of different oncogenic antisense lncRNAs involved in pathways driving HCC, focusing on the best characterized ones (Section 3.1) and dedicating two subsections to the most studied antisense lncRNAs (Section 3.1.1 and Section 3.1.2); then, we look at the antisense lncRNAs that function as tumor suppressors to hinder malignant growth (Section 3.2). An extended list of antisense lncRNAs, their dysregulation, and their molecular functions in HCC is provided in Table 1.

### 3.1. Antisense lncRNAs Function as Oncogenes

Alteration of different molecular pathways is known to be involved in HCC development and progression. Among them, the Wnt/β-catenin signaling pathway is a well-known oncogenic pathway, and its increased activation frequently occurs in HCC. The expression status of β-catenin is a key feature that affects Wnt/β-catenin signaling and plays a key role in signal transduction to activate the transcription of Wnt target genes that are involved in tumor onset and progression [149]. Interestingly, several studies indicate that antisense lncRNAs may play an important role in the regulation of the Wnt/β-catenin pathway. In this regard, a recent study indicates TMPO-AS1 as a novel oncogene in HCC, where it competitively binds to miR-126-3p, thus increasing LRP6 expression to activate the Wnt/β-catenin pathway [116]. This activation can also be mediated by an epigenetic mechanism triggered by another antisense lncRNA, HOXA11-AS1: RIP and RNA pull-down experiments demonstrated that it was able to recruit the DNA methyltransferase DNMT1 to promoter of HOXA11, thus inhibiting its expression and resulting in the activation of the Wnt signaling pathway [25]. Another antisense lncRNA involved in the regulation of the Wnt/β-catenin pathway is SOX9-AS1. SOX9 has been reported to positively regulate β-catenin expression in the canonical Wnt pathway to promote cancer progression [150]. Zhang and colleagues demonstrated that SOX9-AS1 can increase the expression of its sense gene SOX9, sponging miR-5590-3p to activate Wnt/β-catenin signaling [112]. Furthermore, KCNQ1OT1 was found to be upregulated in human HCC tissues and correlated with liver cirrhosis, advanced TNM stage, and large tumor size. KCNQ1OT1 can regulate GSK3β/β-catenin/Bcl-2 signaling pathway, competitively binding to miR-504 [80]. Likewise, OTUD6B-AS1 may accelerate HCC cell proliferation and invasion by sequestering miR-664b-3p to induce GSKIP upregulation, which is associated with the activation of Wnt/β-catenin signaling [99]. 

Another pathway that plays a crucial role in HCC is the Akt/mTOR pathway. The Akt/mTOR signaling pathway regulates crucial cellular processes in the physiological setting as well as most hallmarks of cancer, including cell cycle, survival, metabolism, motility, and angiogenesis [151]. Several studies indicate that antisense lncRNAs may be involved in Akt/mTOR regulation. For instance, ZEB1-AS1 is involved in HCC bone metastasis via the epigenetic suppression of miR-302b expression, resulting in enhanced EGFR/PI3K-AKT signaling [130]. Min and colleagues demonstrated that MYC could activate the transcription of DLG1-AS1 that, in turn, could regulate MYC expression. Thus, the MYC/DLG1-AS1 axis could promote HCC cell growth and migration by activating PI3K/AKT and Src/FAK pathways. Moreover, DLG1-AS1 may boost HCC cell growth and migration by regulating the miR-497-5p/SSRP1 axis [60]. In addition, TMPO-AS1 may exert its oncogenic effect in HCC by sponging miR-329-3p to upregulate FOXK1 and activate the AKT/mTOR signaling pathway [117]. 

Hypoxia is a crucial feature in all solid tumors, especially HCC. Although hypoxia itself is toxic to cancer cells, a hypoxic environment leads HCC cells to induce a series of adaptive, “pro-survival” changes, which subsequently leads to elevated angiogenesis, adapted metabolic alteration, tumor invasion, and metastasis [152,153]. He and colleagues identified NPSR1-AS1 as a novel hypoxia-responsive lncRNA by using microarray analysis. NPSR1-AS1 was found to be highly expressed in HCC tissues, and its overexpression enhanced the proliferation and glycolysis of HCC cells. Previous studies have shown that the MAPK/ERK pathway is activated in HCC cells under hypoxic conditions. Interestingly, the authors found that the MAPK/ERK pathway was positively regulated by NPSR1-AS1 in HCC cells, suggesting that NPSR1-AS1 may promote the proliferation and glycolysis of HCC cells by regulating the MAPK/ERK pathway [97]. Furthermore, in the hypoxic microenvironment, HIF-1α, a master regulator of hypoxia, can induce the expression of ALKBH3-AS1; Lu and colleagues demonstrated that the half-life of ALKBH3 mRNA was reduced after silencing ALKBH3-AS1, suggesting that the antisense lncRNA can exert an oncogenic role by enhancing ALKBH3 mRNA stability in HCC cells [48]. Similarly, NR2F1-AS1 expression is enhanced in HCC under hypoxia. Interestingly, in vitro experiments demonstrated that NR2F1-AS1 knockdown may suppress hypoxia-induced glycolysis and migration in HCC, modulating the miR-140/HK2 axis [98]. Antisense lncRNA USP2-AS1 was found to promote tumor growth under hypoxia conditions; in particular, RIP and RNA pull-down assays demonstrated that USP2-AS1 can interact with YBX1 and significantly increase its binding to HIF-1α mRNA, promoting translation [125].

Beyond the involvement of antisense lncRNAs in the well-known oncogenic pathways described above, many other dysregulated antisense lncRNAs in HCC have been identified in several studies. Among them, LEF1-AS1 is highly expressed in HCC tissues and cells, promoting proliferation and invasion by increasing EZH2 expression via the CEBPB-CDCA7 signaling pathway [30]. Moreover, in vitro and in vivo experiments indicate that the LEF1-AS1 may be responsible for the progression of HCC by sponging miR-136-5p and thus upregulating WNK1 expression [83]. Another example is provided by ZEB2-AS1, which is involved in HCC cell proliferation and metastasis through the expression modulation of its sense gene ZEB2, which plays a central role in epithelial–mesenchymal transition (EMT) [154]. Consistent with this, another study demonstrated the oncogenic potential of ZEB2-AS1 by targeting the miR-582-5p/FOXC1 axis [131]. Finally, an intriguing ceRNA network (ceRNET) contributing to heaptocarcinogenesis involves the oncosuppressive miR-125a and let-7e, their mRNA targets, and SPACA6P-AS, an lncRNA transcribed from the same miRNAs locus but in the opposite direction, and thus carrying complementary sequences to the miRNAs. In brief, SPACA6P-AS, upregulated in HCC tissues, can sponge miR-125a and let-7e, thus reducing their silencing activity toward key oncogenic targets, such as Lin28b, MMP11, SIRT7, Zbtb7a, Cyclin D1, CDC25B, and HMGA2 [113].

#### 3.1.1. HOTAIR

One of the most well studied antisense lncRNA in HCC is the HOX transcript antisense RNA (HOTAIR), which is transcribed from the HOXC locus and interacts with both the polycomb repressive complex 2 (PRC2) and lysine-specific histone demethylase 1A complex to enhance histone H3 lysine trimethylation and histone H3 lysine 4 demethylation, leading to the silencing of multiple genes [155]. Recently, the upregulation of HOTAIR in HCC was observed in several studies, and its expression in patients was associated with clinicopathological features such as metastasis and tumor size, and correlated with poor prognosis [156]. 

At the cellular level, HOTAIR is involved in proliferation, cell motility, invasion, cell cycle progression, apoptosis, and autophagy through a plethora of molecular mechanisms summarized here. 

Upregulation of HOTAIR can promote the proliferation, migration, and invasion of human HCC cells by regulating ATG3 and ATG7 expression, which are involved in autophagosome formation and activation [69]. Ding and colleagues demonstrated that HOTAIR increases HCC cell invasion by suppressing RBM38 expression, which plays a role in regulating cell motility [70]. In the HCC cell lines, the knockdown of HOTAIR suppresses cell proliferation and invasion by inhibiting the Wnt/β-catenin signaling pathway [71]. Additionally, HOTAIR knockdown can regulate the G1/S phase transition of the cell cycle by inhibiting STAT3 phosphorylation and reducing cyclin D1(CCND1) expression [72]. Aberrant fucosylation, which plays an important role in HCC metastasis, can also be modulated by HOTAIR alteration through the HOTAIR/STAT3/FUT8/MUC1 feedback loop via the JAK1/STAT3 cascade. Mechanistically, HOTAIR can recruit P300 to efficiently bind to STAT3; then, the transcriptional complex comodulate the transcription of FUT8 and MUC1 [32]. Furthermore, HOTAIR can promote glycolysis in HCC cells, indicating that is involved in the metabolic processes. Wei and colleagues demonstrated that HOTAIR can increase GLUT1 expression by upregulating mTOR; moreover, RIP assays demonstrated that HOTAIR could directly bind to GLUT1, thus enhancing its stability [73]. 

HOTAIR has also been reported to exploit the ceRNA mechanism to regulate tumor progression [157]. In this regard, under hypoxia, HOTAIR can promote glycolysis upregulating HIF-1α by sponging miR-130a-3p [74]. Moreover, Su and colleagues demonstrated that HOTAIR expression is regulated by FOXC1, and that its oncogenic activity is partly based on miR-1 sponging [75]. HOTAIR may also sponge miR-214-3p to upregulate FLOT1, resulting in increased cell proliferation, migration, and invasion [76].

#### 3.1.2. ANRIL

Another well-studied antisense lncRNA is the antisense non-coding RNA from the INK4 locus (ANRIL), also known as CDKN2B-AS. ANRIL is transcribed as a 3.8 kbp lncRNA that contains 19 exons in the antisense direction of the INK4B-ARF-INK4A gene cluster at chromosome 9p21 and is involved in its repression. ANRIL was found significantly upregulated in HCC tissues and cells, suggesting that it may play an important role in HCC progression [158], with multiple molecular mechanisms discussed here. 

Huang and colleagues proposed that ANRIL could regulate cell growth, both in vitro and in vivo, via epigenetic silencing of Kruppel-like factor 2 (KLF2) by binding to PRC2 [49]. ANRIL knockdown significantly decreases the activation of the AKT/mTOR pathway in HCC cells via the let-7c-5p/NAP1L1 axis [50]. Moreover, ANRIL knockdown may suppress proliferation, migration, and invasion, and promote apoptosis in HepG2 cells by sponging miR-191, leading to the inactivation of NF-κB and Wnt/β-catenin signaling pathways [51]. Further, ANRIL is involved in the regulation of MEK/ERK signaling. Chen and colleagues demonstrated that ANRIL reduces the expression of KLF13, a potential negative regulator of the MEK/ERK signaling pathway, thus inhibiting apoptosis. Furthermore, they highlighted that ANRIL can act as a ceRNA by competitively binding to miR-153-5p, thus increasing ARHGAP18 expression to promote tumorigenesis and metastasis in HCC [52]. Moreover, a recent study revealed that ANRIL may regulate mitochondrial functions by acting as a competing endogenous RNA to increase ARL2 expression via sponging miR-199a-5p, highlighting a novel mechanism by which ANRIL can regulate HCC progression [53].

### 3.2. Antisense lncRNAs Function as Tumor Suppressor

The vast majority of antisense lncRNAs with a role in HCC have been characterized as oncogenes; a few other antisense lncRNAs have been reported as tumor suppressors. 

One example of this is represented by the lncRNA MAGI2-AS3. In vitro, MAGI2-AS3 is able to impede cell proliferation, invasiveness, and migration and enhance cell apoptosis; in vivo, MAGI2-AS3 remarkably restrained tumorigenesis in an HCC mouse model. Mechanistically, MAGI2-AS3 can decrease the expression of the oncogene RACGAP1 by inducing histone demethylation of its promoter through recruitment of the demethylase KDM1A, as demonstrated by Chip in RIP and RNA pull-down assays, [144]. MAGI2-AS3 may also affect HCC progression by sponging miR-519c-3p, which is known to play a role in HCC growth and metastasis in vitro and in vivo. Interestingly, TXNIP, a regulator of tumor microenvironment, is a target of miR-519c-3p, highlighting a new ceRNA mechanism in the functions of MAGI2-AS3 [143]. 

Likewise, WWOXA-S1 is downregulated in HCC tissues and cell lines, and is involved in the proliferation, apoptosis, migration, and EMT processes. Interestingly, WWOX, the sense gene of WWOX-AS1, has been confirmed to exert an anticancer role in various cancers and is found to be downregulated in HCC. Xu and colleagues demonstrated that WWOX-AS1 may function as a ceRNA by sequestering miR-20b-5p, thus upregulating WWOX expression [148]. 

In addition, the lncRNA ADORA2A-AS1 may exert a tumor-suppressive role in HCC. Low expression of ADORA2A-AS1 in HCC tissues correlates with advanced stage, invasion, and poor survival rate. At the cellular level, ADORA2A-AS1 can repress proliferation, migration, and invasion; it is also able to restrict HCC xenograft growth and metastasis in vivo. Mechanistically, ADORA2A-AS1 can interact with HuR and suppress the binding of HuR to its mRNA target, FSCN1, resulting in the inhibition of the Akt pathway [139]. 

Abnormal activation of the hedgehog signaling pathway is one pivotal cause of cancer initiation and progression [159]. HHIP is a negative regulator of the hedgehog signaling pathway and exhibits suppressive effects in cancers [160]. Bo and colleagues proposed that HHIP-AS1 may modulate the hedgehog signaling pathway via HHIP in a post-transcriptional manner. In this regard, an RNA pull-down assay showed that HHIP-AS1 interacts with and stabilizes HHIP mRNA; then, RIP assays demonstrated that HHIP-AS1 may promote HHIP mRNA stability by facilitating HuR/HHIP interaction [45].

As stated in the Introduction, antisense lncRNAs can interact with other RNA molecules (especially miRNAs) and proteins, but they can also interact with DNA sequences to exert their regulatory role. One example of this, found in HCC, is represented by the tumor suppressor WT1-AS, which has a binding site in the promoter region of WT1; this binding, demonstrated by reporter constructs and WT1-AS overexpression, downregulated the expression of WT1, thus promoting cell apoptosis by suppressing the JAK/STAT3 signaling pathway [147]. 

## 4. Perspectives in Diagnostics and Therapeutics 

The knowledge of the molecular mechanisms underlying the role of antisense lncRNA in HCC may pave the way to new perspectives on their clinical use, both as innovative diagnostic/prognostic biomarkers and as new therapeutic targets.

In this section, we will first discuss their possible use as biomarkers, providing some relevant examples; then, we will focus on their potential as targets for HCC treatment by providing an overview of possible antisense lncRNA-based therapies.

### 4.1. Diagnostic and Prognostic Antisense lncRNAs 

**Figure 3:**
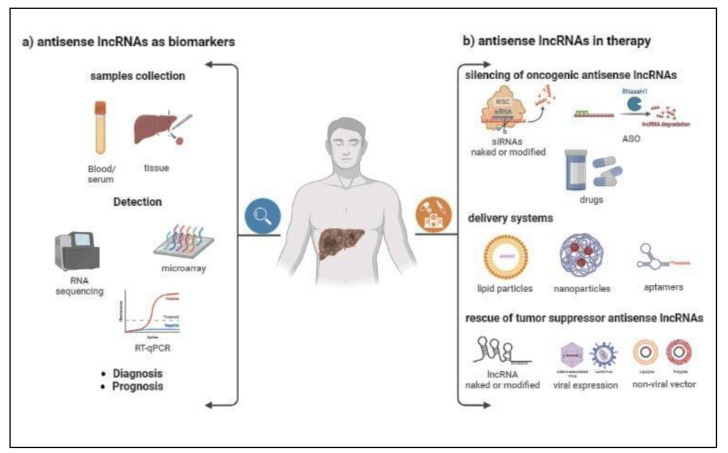
Perspectives in diagnostics and therapeutics of antisense lncRNAs. (**a**) Antisense lncRNAs purified from liquid biopsies or tissues could be analyzed by RNA sequencing, microarray, or RT-qPCR arrays, and their expression levels may serve as potential biomarkers for HCC diagnosis and prognosis. (**b**) Small interfering RNA (siRNA), antisense oligonucleotide (ASO), and small molecules could target oncogenic antisense lncRNAs and downregulate their expression. These could be introduced into the cells using various delivery systems. Moreover, the expression of tumor suppressor antisense lncRNAs could be restored by using synthetic lncRNAs, and with viral vectors or nonviral vectors driving their overexpression. Figure created with BioRender.com.

Despite the recent progress in HCC diagnosis, the overall prognosis still remains unsatisfactory, and there is an urgent need to search for new biomarkers with high efficacy for the early detection of HCC and prediction of tumor progression [161]. The identification of antisense lncRNAs whose expression levels correlate with clinicopathological characteristics of patients sheds new light on their potential diagnostic and/or prognostic role in HCC. In addition, some antisense lncRNAs have been found in the extracellular space, not only contributing to tumoral microenvironment, but also providing the chance to exploit their dosage for diagnostic/prognostic purposes by a noninvasive approach such as serum samples collection (Figure 3a).

For instance, HOTAIR, an oncogenic lncRNA significantly overexpressed in HCC tissues, was found to be associated with an increased risk of recurrence after hepatic resection; this risk was significantly higher in patients with lymph node metastasis [162]. Another study demonstrated that serum HOTAIR was clearly overexpressed in patients with HCC compared to cirrhotic patients and healthy subjects, and was correlated with advancement of HCC stage. Thus, the circulating HOTAIR may be used as a valuable clinical biomarker for HCC diagnosis and prognosis of the clinical outcome [163]. Consistently, the serum level of HOTAIR was observed to be significantly decreased following surgical resection in HCC patients. Hence, HOTAIR, together with the lncRNA ICR, may be a useful marker for monitoring the outcome of surgical resection in HCC patients [164]. Moreover, El-Khazragy and colleagues showed that HOTAIR may be a biological marker to predict the risk of developing HCC following DAAs (direct-acting antivirals) in HCV-related cirrhosis, allowing for the selection of patients at higher risk of developing HCC after DAAs treatment [165].

Similarly, high expression levels of ANRIL [166], HOXB-AS1 [167], HOXC13-AS [168], DDX11-AS1 [169], ELF3-AS1 [170], FOXP4-AS1 [171], PITPNA-AS1 [172], MAFG-AS1 [173], ROR1-AS1 [174], and ZFAS1 [175], as well as the downregulation of ZNF385D-AS2 [176], have been variably shown to be correlated with pathological features and clinical outcome as survival and response to pharmacological treatment of HCC patients (Table 2).

The real future challenge may be the design of a panel combining the dosage of the molecules independently identified in the various studies to further validate them with the same methodologies in large cohorts of patients and, ultimately, select those antisense lncRNAs endowed with diagnostic/prognostic potentiality and clinical utility.

### 4.2. Role of Antisense lncRNAs in Chemoradioresistance

Although the clinical treatment of HCC has vastly improved, the five-year survival rate of HCC patients is still low because of high refractoriness to chemotherapy. This is due to the interaction of very complex and diverse mechanisms of chemoresistance (MOC) that protect tumor cells from the chemotherapeutic [179]. Recently, increasing evidence indicates that antisense lncRNAs are involved in drug-resistant processes, suggesting new strategies to solve this problem [11]. Below, we discuss some antisense lncRNAs whose role and molecular mechanisms in drug resistance have been determined (Table 3).

Sorafenib is a multitarget kinase inhibitor that suppresses tumor cell proliferation and angiogenesis. Its mechanism of action was also related to ncRNAs in HCC, e.g., to the oncosuppressive miR-125a [187]. Sorafenib was approved by the FDA in 2007 as a unique target drug for advanced HCC. However, its treatment efficacy is affected by the frequent occurrence of drug resistance [188]. Tang and colleagues found that HCC cells with higher HOTAIR expression exhibited greater resistance to sorafenib and that HOTAIR knockdown was able to inhibit the proliferation of HCC cells in the presence of sorafenib. Subsequently, the authors found that HOTAIR may negatively regulate the expression of miR-217 thus promoting EMT, thus inducing sorafenib resistance [182]. Furthermore, MAFG-AS1 knockdown may inhibit the proliferation, migration, invasion, and tumor growth of sorafenib-resistant HCC cells in vitro, suggesting the potential role of MAFG-AS1 in tumor-promoting drug resistance. Mechanistically, MAFG-AS1 could target and negatively regulate miR-3196 to promote STRN4 expression in order to induce sorafenib resistance [185].

Cisplatin (CDDP) is a drug extensively administered for the treatment of various cancers, including HCC. Thus, it is imperative to determine the molecular mechanisms underlying HCC chemoresistance to aid the diagnosis and treatment of patients with HCC. Multidrug resistance (MDR) could be caused by the overexpression of ATP-binding cassette subfamily B member 1 (ABCB1), which pumps anticancer agents out of cells, contributing to a reduced intracellular drug concentration and cytotoxicity. Inhibition of ABCB1 expression is able to reduce the resistance of HCC cells to anticancer agents [189]. LncRNA HOTAIR could mediate cisplatin resistance of HCC cells by regulating ABCB1 expression through STAT3 activation, identifying HOTAIR as a potential novel therapeutic target to reverse MDR in HCC [181]. Gao and colleagues demonstrated that LEF1-AS1 is elevated in HCC cells, and silencing LEF1-AS1 impeded proliferation and promoted the apoptosis of CDDP-resistant HCC cells. At the molecular level, LEF1-AS1 could amplify the chemoresistance of HCC cells by functioning as a miR-10a-5p regulator to enhance MSI1 expression and induce the AKT signaling pathway [184]. Moreover, FGD5-AS1 could be involved in CDDP resistance in HCC cells. FGD5-AS1 was found to increase in CDDP-resistant HCC tissues and cells, and its silencing may decrease the IC50 value of CDDP, cell viability, and invasion, and facilitate cell apoptosis. FGD5-AS1 is able to modulate TWF1 expression to induce CDDP resistance in HCC cell lines by targeting miR-153-3p [180].

Similarly, radiation therapy is a very common treatment for many types of cancer, either alone or in combination with other therapeutic methods. Its effectiveness greatly depends on the radiosensitivity of the cancer cells. Some studies reported that dysregulation of antisense lncRNAs might be involved in this process. For instance, in HCC, lncRNA TP73-AS1 is highly expressed and increases after X-ray irradiation. Song and colleagues demonstrated that TP73-AS1 overexpression may induce pAkt/Akt expression, leading to a reduction of PTEN expression. Thus, lncRNA TP73-AS1 silencing may repress the development of HCC and promote radiosensitivity in HCC via the PTEN/Akt signaling pathway, improving HCC therapy [186].

### 4.3. Antisense lncRNAs in Therapy

As discussed, antisense lncRNAs have critical roles in HCC onset and progression. Thus, their modulation may be a promising tool for HCC treatment. In recent years, great progress was achieved in developing biological drugs that target RNA molecules in cells, providing the basis for lncRNA-based cancer therapies. These therapies are essentially based on the silencing of oncogenic antisense lncRNA or, ideally, on restoring the expression of tumor suppressor lncRNAs (Figure 3b). Of these two fields, the first has been explored more because the vast majority of antisense lncRNAs act as an oncogene (Table 1).

In this regard, RNA interference (RNAi) has been studied and developed extensively and represents a powerful method to target RNA molecules in cells. RNAi can be triggered experimentally by exogenous introduction in the cells of double-stranded RNAs (dsRNAs) or constructs which express short-hairpin RNA (shRNAs). Small interfering RNAs (siRNAs) are double-stranded RNAs that are usually approximately 20 base pairs long. They can interfere with the expression of specific genes by degrading target RNA and/or preventing translation. Once introduced into the cells, siRNAs are bound by RISC (RNA-induced silencing complex), the RNA duplex is separated, and the strand with the less stable 5′-end is loaded onto the active RISC complex. Then, the RISC complex can recognize the target transcript, leading to its cleavage, and resulting in gene silencing [190]. Thus, siRNAs could be used to inhibit antisense lncRNAs in cells. For instance, the knockdown of HOTAIR by siRNA can reduce proliferation, migration, and invasion of human HCC cells [69] and inhibit tumor growth in an HCC mouse xenograft model [76]. Although the therapeutic use of siRNAs has long been evaluated in different phases of clinical trials, the main issue remains the half-life and the delivery of siRNAs. For systemic administration, siRNAs must be stable in circulation and resistant to degradation. In this regard, several studies indicated that chemical modifications may improve siRNAs’ stability. For instance, the incorporation of tryptamine and diphenylpropylamine at the 3′-end of siRNAs may improve silencing activity and increase stability in serum [191]. Further, the modification of siRNAs containing terminal amide linkages, by introducing hydroxyethylglycine PNA (hegPNA), can improve their biostability, markedly increasing the resistance to serum-derived nucleases [192]. Moreover, the use of nanoparticles and lipid-based delivery tools may improve the delivery and uptake of siRNAs [193,194]. Finally, extensive secondary structures along the antisense lncRNA molecules may represent another obstacle. However, aptamers can easily overcome this obstacle [195]. For instance, a chimeric aptamer coupled with the siRNA of HOTAIR can deliver the siRNA into the cells and suppress cell growth and invasion [196]. The next challenge could be the delivery of therapeutic RNAs to cancer cells for preventing off-target effects; however, this is a general goal in the therapeutic field.

Antisense oligonucleotides (ASOs) are small-sized, single-stranded nucleic acids that can target both nuclear and cytoplasmic-located antisense lncRNAs. Usually, RNaseH1 recognizes the DNA:RNA heteroduplex and catalyzes the RNA cleavage [197]. As mentioned before, ANRIL is known to play an oncogenic role in HCC and its depletion could be a therapeutic option. Yap and colleagues demonstrated that two different ASOs against ANRIL can reduce cell proliferation by blocking critical elements of ANRIL [198]. Similarly to siRNAs, the stability and selectivity of ASOs have been greatly improved by various chemical modifications. For instance, gapmers contain a central DNA part, which activates RNase H-mediated RNA degradation, flanked by modified oligonucleotides, such as 2′-O-methyl RNA (2′OMe), and locked nucleic acids (LNAs) which increase target affinity and resistance to nuclease activity. Furthermore, LNA gapmers have been shown to enable an unassisted delivery method, making them superior to siRNAs, for which the formulation of the transport vehicle is critical for effective in vivo delivery [195]. 

The above strategies exploit the versatility and increasing knowledge of ncRNA biology by using different RNA-based tools targeting other RNA molecules. The discovery that some drugs may also exert their anticancer activity by regulating antisense lncRNAs could suggest additional therapeutic interventions, e.g., based on the combination of therapeutic RNAs and drugs. For instance, Tian and colleagues demonstrated that curcumol could downregulate HOTAIR expression, which induces the suppression of EZH2, leading to the inhibition of tumor growth [199]. Another study demonstrated that ginsenoside Rg3 significantly inhibits cell proliferation and metastasis by inhibiting the HOTAIR/PI3k/AKT axis [200]. Sevoflurane (SEVO), an anesthetic agent widely applied in clinical settings and used for treating various human cancers, may represent another possibility for the suppression of HCC progression. Interestingly, SEVO treatment is able to downregulate the expression of the oncogenic lncRNA KCNQ1OT1 in HCC cell lines [201].

Overall, the studies reported above show the great potential of antisense lncRNA-targeted HCC therapies, encouraging further studies for deepening the molecular mechanisms and ultimately assessing specificity, tolerable side effects, safety, formulations, and delivery. 

## 5. Conclusions

Recent insights into RNA biology induced a paradigm shift towards the recognition of RNAs—beyond their role of serving as messengers for protein-encoding genes—as functional molecules. In this new perspective, DNA can be envisaged as the “hardware”, and RNA can be viewed as the “software” managing multiple “biological apps” through its structural/functional versatility. 

Among the different ncRNA biotypes, antisense lncRNAs are emerging as new players in gene expression regulation by exploiting different molecular mechanisms shared with other ncRNA molecules, and by exploiting special molecular mechanisms on their sense gene, through epigenetic, transcriptional, post-transcriptional, and translational modulations. High-throughput sequencing technologies are allowing the annotation of an increasing number of new antisense lncRNAs, found in normal and/or tumor tissues, unveiling various sense–antisense pairs transcribed from several loci. These pairs are no longer considered an oddity but are viewed as a possible evolutionary opportunity to increase complexity and lineage-specific regulatory outcomes. Antisense lncRNAs dysregulation has been shown to be deeply involved in hepatocarcinogenesis, where they can act as an oncogenes or an oncosuppressors, thus playing a key role in tumor onset, progression, and chemoradiotherapy response. The next challenges will be to piece together the entire RNA regulatory networks of antisense lncRNAs and assign them a function in physiological and pathological contexts, and to define prospective novel therapeutic targets and innovative diagnostic tools.

## Figures and Tables

**Table 1:** Antisense lncRNAs acting as oncogenes or tumor suppressors in HCC.

Antisense lncRNA	Role	Effect	Molecular Mechanism	Reference
**AIRN**	Oncogene	Promotes proliferation and inhibits apoptosis	Inhibits CUL4A-mediated ubiquitination of STAT1	[47]
**ALKBH3-AS1**	Oncogene	Promotes cell invasion and proliferation	Enhances ALKBH3 mRNA stability	[48]
**ANRIL**	Oncogene	Associated with clinical outcomes; promotes proliferation, migration and invasion; promotes tumor growth and metastasis in vivo; enhances mitochondrial function	Silences epigenetically Kruppel-like factor 2 (KLF2) by binding to PRC2 sponges let-7c-5p to upregulate NAP1L1, thus activating AKT/mTOR pathway; sponges miR-191, inactivating NF-κB and Wnt/β-catenin pathways; sponges miR-153-5p to upregulate ARHGAP18 and activate MEK/ERK signaling; sponges miR-199a-5p to upregulate ARL2; sponges miR-122-5p	[49,50,51,52,53,54]
**BACE1-AS**	Oncogene	Promotes cell cycle progression, migration, and invasion	Sponges miR-214-3p to upregulate APLN expression	[55]
**BAIAP2-AS1**	Oncogene	Promotes proliferation and metastasis	Sponges miR-361-3p to release SOX4	[56]
**BSG-AS1**	Oncogene	Correlates to hypoxia; promotes proliferation and migration	Enhances the stability of BSG mRNA	[57]
**DARS-A1**	Oncogene	Correlates with poor prognosis; promotes proliferation, cell invasion, and EMT	Sponges miR-3200-5p to upregulate CKAP2 and activate the FAK/ERK pathway	[58]
**DDX11-AS1**	Oncogene	Promotes proliferation, migration, invasion and glucose metabolism	Sponges miR-195-5p to upregulate MACC1 expression	[59]
**DLG1-AS1**	Oncogene	Promotes proliferation, migration, and invasion in HCC and tumor growth in vivo	Induced by MYC; sponges miR-497-5p to upregulare SSRP1	[60]
**DLGAP1-AS1**	Oncogene	Promotes proliferation	Sponges miR-486-5p to upregulate H3F3B	[61]
**DNAJC3-AS1**	Oncogene	Correlates with prognosis of patients; promotes proliferation	Suppresses miR-27b maturation	[62]
**DLX6-AS1**	Oncogene	Promotes cell viability, invasion, and migration	Sponges miR-513c to upregulate Cul4A, thus repressing ANXA10 degradation; sponges miR-424-5p to upregulate WEE1	[63,64]
**FAM83H-AS1**	Oncogene	Associated with tumor prognosis; promotes proliferation, migration, and invasion	Inhibits the Wnt/β-catenin pathway by reducing β-catenin and WNT1 expression	[65]
**FGFR3-AS1**	Oncogene	Promotes proliferation, migration, and invasion; promotes tumor growth in vivo	Activates the PI3K/AKT pathway	[66]
**FOXP4-AS1**	Oncogene	Associated with poor survival, promotes tumor growth in vivo	Recruits EZH2 to the promoter region of ZC3H12D to mediate H3K27me3 methylation, thus inhibiting ZC3H12D expression	[29]
**GATA3-AS1**	Oncogene	Promotes cell proliferation and metastasis	Suppresses PTEN, CDKN1A, and TP53	[67]
**GPC3-AS1**	Oncogene	Indicates poor prognosis; proliferation and migration; Promotes xenograft tumor growth in nude mice	Recruits PCAF to the GPC3 gene body region, upregulating GPC3 transcription	[68]
**HOTAIR**	Oncogene	Promotes proliferation, migration, invasion, and tumor growth in vivo; regulates the G1/S phase transition; regulates glycolyis; associated with poor survival rates	Increases ATG3 and ATG7 expression; inhibits RBM38; activates Wnt/β-catenin pathway, increases CCND1 expression and STAT3 signaling; binds STAT3 andP300 to upregulate FUT8 and MUC1; upregulates GLUT1, upregulating mTOR; sponges miR-130a-3p to upregulate HIF1A regulated by FOXC1; sponges miR-1; sponges miR-214-3p to upregulate FLOT1	[32,69,70,71,72,73,74,75,76]
**HOXA11-AS1**	Oncogene	Promotes proliferation, invasion, and self-renewal	Suppresses the transcription of HOXA11 by recruiting DNMT1 to the promoter activating Wnt/βcatenin pathway	[25]
**HOXA-AS2**	Oncogene	Promotes cell migration and invasion by inducing EMT	Sponges miR-520c-3p to upregulate GPC3	[77]
**HOXD-AS1**	Oncogene	Promotes proliferation and invasion; regulates cell cycle progression	Sponges miR-miR-326 to upregulate SLC27A4, induces MEK/ERK signaling pathway	[78,79]
**KCNQ1OT1**	Oncogene	Correlates with liver cirrhosis, an advanced TNM stage, and a large tumor size; promotes proliferation and tumor growth in vivo	Sponges miR-504 to regulate GSK3β/β-catenin/Bcl-2 signaling pathway	[80]
**KTN1-AS1**	Oncogene	Associated with poor survival, promotes proliferation	Sponges miR-23c to upregulate ERBB2IP	[81]
**LASP1-AS**	Oncogene	Associated with poor prognosis, enhances proliferation and migration	Upregulates LASP1	[82]
**LEF1-AS1**	Oncogene	Promotes proliferation, invasion, angiogenesis, and tumor growth in vivo	Sponges miR-136-5p to regulate WNK1 espression, recruits CEBPB to promote CDCA7/EZH2 expression	[30,83]
**LOXL1-AS1**	Oncogene	Promotes proliferation, migration, and invasion	Sponges miR-3614-5p to upregulate YY1	[84]
**MACC1-AS**	Oncogene	Increases stemness; promotes cell proliferation, EMT, and invasion	Sponges miR-145 to regulate Nanog, Oct4, and Sox9; regulates PAX8	[85,86]
**MAFG-AS1**	Oncogene	Promotes proliferation, invasion, and migration	Sponges miR-6852	[87]
**MAPKAPK5-AS1**	Oncogene	Associated with poor clinical features and prognosis, promotes growth and metastasis	Sponges miR-154-5p to upregulate PLAGL2, thus activating EGFR/AKT signaling and regulating HIFA	[88]
**MCM3AP-AS1**	Oncogene	Correlated with poor prognosis, promotes cell growth	Sponges miR-194-5p to upregulate FOXA1	[89]
**MFI2-AS1**	Oncogene	Promotes invasion and metastasis of HCC cells in vitro and vivo	Sponges miR-134 to upregulate FOXM1 expression	[90]
**MKLN1-AS**	Oncogene	Promotes proliferation, migration, invasion, and tumor growth in vivo; associated with poor prognosis	Sponges miR-22-3p to upregulate ETS proto-oncogene 1, sponges miR-654-3p to upregulate HDGF	[91,92]
**MYLK-AS1**	Oncogene	Associated with poor prognosis; promotes cell invasion, migration, proliferation, and angiogenesis	Sponges miR-424-5p to upregulate E2F7 and activate VEGFR2 signaling; increases EGFR, pEGFR, HER2 and RAF1 expression	[93,94]
**NNT-AS1**	Oncogene	Decreases CD4 lymphocyteinfiltration, promotes proliferation in vitro and tumor growth in vivo	Enhances TGF-β signaling pathway, sponges miR-363 to upregulate CDK6 expression	[95,96]
**NPSR1-AS1**	Oncogene	Promotes proliferation and glycolysis	Regulates MAPK/ERK pathway	[97]
**NR2F1-AS1**	Oncogene	Induces glycolysis under hypoxia and promotes migration	Sponges miR-140 to upregulate HK2	[98]
**OTUD6B-AS1**	Oncogene	Promotes proliferation and invasion	Sponges miR-664b3-p to induce GSKIP/Wnt/β-catenin signalling	[99]
**PCNA-AS1**	Oncogene	Promotes tumor growth in vitro and in vivo	Stabilizes PCNA transcripts	[100]
**PITPNA-AS1**	Oncogene	Promotes proliferation, migration, and EMT	Sponges miR-876-5p to upregulate WNT5A	[101]
**PRKAG2-AS1**	Oncogene	Associated with poor survival rates; promotes proliferation, migration, and invasion	Sponges miR-502-3p to upregulate BICD2	[102]
**PRR34-AS1**	Oncogene	Promotes proliferation migration, invasion, and EMT; enhances tumor growth in vivo	Sponges miR-296-5p to upregulate E2F2 and SOX12, activating Wnt/βcatenin pathway; interacts with DDX3X to regulate the stability of Rab27a mRNA and promote the exosome secretion of VEGF and TGF-β; sponges miR-498 to upregulate TOMM20 and ITGA6	[103,104,105]
**RBM5-AS1**	Oncogene	Promotes cell proliferation and invasion	Sponges miR-132/212 via recruiting PRC2 complex	[106]
**RHPN1-AS1**	Oncogene	Correlated with prognosis of patients; promotes proliferation and metastasis; associated with the occurrence of lymphatic metastasis and a higher level of serum AFP; correlated with poor survival	STAT1 induces overexpression of RHPN1-AS1, sponges miR-485 to upregulate CDCA5, sponges miR-596 to upregulate IGF2BP2	[107,108]
**RNF185-AS1**	Oncogene	Correlated with advanced TNM stage, distant metastasis and a poor survival rate; promotes proliferation, migration, and invasion	Sponges miR-221-5p to upregulate ITGB5	[109]
**SBF2-AS1**	Oncogene	Correlated with poor prognosis, promotes proliferation and tumor growth in vivo	Sponges miR-140-5p to upregulate TGFBR1 expression	[110]
**SNAI3-AS1**	Oncogene	Promotes proliferation and metastasis	Sponges miR-27-3p/34a-5p	[111]
**SOX9-AS1**	Oncogene	Promotes proliferation, migration, and invasion; Promotes tumor growth and metastasis in vivo	Sponges miR-5590-3p to upregulate SOX9, thus activating Wnt/b-catenin pathway	[112]
**SPACA6P-AS**	Oncogene	Promotes cell proliferation	Sponges miR-125a/Let7a to upregulate Lin28b, MMP11, SIRT7, Zbtb7a, Cyclin D1, CDC25B, HMGA2	[113]
**ST8SIA6-AS1**	Oncogene	Promotes proliferation, migration, and invasion	Sponges miR-338-3p to upregulate NONO expression, sponges miR-5195-3p to regulate HOXB6 expression	[114,115]
**TMPO-AS1**	Oncogene	Associated with poor prognosis, promotes proliferation and EMT	Sponges miR-126-3p to upregulate LRP6, inducing Wnt/β-catenin signalling; sponges miR-329-3p to upregulate FOXK1, inducing AKT/mTOR signaling pathway; sponges miR-320a to upregulate SERBP1	[116,117,118]
**TP73-AS1**	Oncogene	Correlated with poor prognosis, promotes proliferation	Sponges miR-200a to induce HMGB1/RAGE pathway	[119]
**TRG-AS1**	Oncogene	Promotes proliferation, migration, invasion, and EMT progress	Sponges miR-4500 to modulate BACH1	[120]
**TRIM52-AS1**	Oncogene	Promotes proliferation and EMT	Sponges miR-514a-5p to upregulate MRPS18A	[121]
**TTN-AS1**	Oncogene	Promotes proliferation, migration, and EMT	Sponges miR-139-5p to upregulate SPOCK1 expression	[122]
**UNC5B-AS1**	Oncogene	Promotes proliferation, migration, and EMT	Sponges miR-4306 to upregulate KDM2A expression	[123]
**UPK1A-AS1**	Oncogene	Correlated with poor prognosis, promotes proliferation and cell cycle progression	Interacts with EZH2; sponges miR-138-5p	[124]
**USP2-AS1**	Oncogene	Increases proliferation, migration, and invasion under hypoxia	Interacts with YBX1 to increase the protein translation of HIF1a under hypoxia	[125]
**VPS9D1-AS1**	Oncogene	Facilitates cell proliferation, migration, and stemness	Sponges miR-491-5p to upregulate SEC61A1	[126]
**WEE2-AS1**	Oncogene	Positively correlated to HBV infection; increases proliferation, migration, invasion, and cell cycle progression	Upregulates FERMT3 expression and activates PI3K/AKT/GSK3b signaling	[127]
**ZEB1-AS1**	Oncogene	Promotes proliferation and invasion, associated with bone metastasis	Sponges miR-229-3p to upregulate E2F1 expression, sponges miR-23c, sponges miR-302b to increase PI3K-AKT pathway activation and EGFR expression	[128,129,130]
**ZEB2-AS1**	Oncogene	Associated with large tumor volume, increased tumor-node-metastasis (TNM) stage, and positive lymph node metastasis; promotes proliferation, migration, invasion, and suppressed apoptosis	Sponges miR-582-5p to upregulate FOXC1	[131]
**ZFAS1**	Oncogene	Associated with worse prognosis and survival; promotes proliferation, migration, and invasion	Sponges miR-624 to upregulate MDK-mediated ERK/JNK/AKT signaling pathway	[132]
**ZFPM2-AS1**	Oncogene	Correlated with advanced TNM stage, distant metastasis, and a poor survival rate; promotes proliferation, migration, invasion, and tumor growth in vivo	Sponges miR-1226-3p to upregulate ITGB1, sponges miR-3065-5p activity to regulate XRCC4, sponges miR-139 to upregulate GDF10, sponges miR-653 to upregulate GOLM1	[133,134,135,136]
**ZSCAN16-AS1**	Oncogene	Correlated with poor clinical outcomes; promotes proliferation, migration, and invasion	Sponges miR-451a to increase ATF2 expression; sponges miR-181c-5p to upregulate SPAG9, activating JNK	[137,138]
**ADORA2A-AS1**	Tumor suppressor	Inhibits proliferation, migration, and invasion; represses xenograft growth and metastasis in vivo	Competitively binds HuR decreasing FSCN1 transcript stability, thereby repressing the AKT pathway	[139]
**CADM1-AS1**	Tumor suppressor	Inhibits proliferation, migration, invasion, and tumor growth in vivo	Regulates the AKT/GSK-3β signaling pathway	[140]
**F11-AS1**	Tumor suppressor	Suppresses proliferation, migration, and invasion	Sponges miR-221-5p to upregulate NR1I3	[141]
**HHIP-AS1**	Tumor suppressor	Downregulation of HHIP-AS1 correlates with larger tumor size, metastasis, and advanced TNM stage; inhibits proliferation, migration, and invasion; induces apoptosis	Facilitates HHIP mRNA stability by promoting HuR binding to HHIP mRNA	[45]
**HNF1A-AS1**	Tumor suppressor	Suppresses proliferation, migration, and invasion; inhibits tumorigenesis and metastasis in vivo	Interacts and activates SHP-1	[142]
**MAGI2-AS3**	Tumor suppressor	Inhibits proliferation in vitro and tumor growth in vivo	Sponges miR-519c-3p to increase TXNIP, decreases RCGAP1 expression by facilitating histone demethylation of the RACGAP1 promoter by recruiting KDM1A	[143,144]
**TMEM220-AS1**	Tumor suppressor	Suppresses proliferation and invasion	Increases TMEM220 expression to regulate Wnt/β-catenin pathway	[145]
**UCHLAS1**	Tumor suppressor	Inhibits proliferation and migration	Enrichment analysis reveals that HRAS, BMP4, and CALM3 are hub genes of HCC, related to UCHLI-AS1	[146]
**WT1-AS**	Tumor suppressor	Promotes cell apoptosis	Inhibits JAK2/STAT3 and MAPK signaling, regulates WT1 by binding promoter region	[147]
**WWOX-AS1**	Tumor suppressor	Decreases cell proliferation, migration and EMT	Sponges miR-20b-5p to upregulate WWOX expression	[148]

**Table 2:** Antisense lncRNAs as potential prognostic and diagnostic biomarkers.

Antisense lncRNA	Clinical Utility	Sample Source	Reference
**ANRIL**	Prognosis	Tissue	[166]
**DDX11-AS1**	Diagnosis, prognosis	Tissue	[169]
**ELF3-AS1**	Diagnosis, prognosis	Tissue	[170]
**ELMO1-AS1**	Prognosis	Tissue	[177]
**FOXP4-AS1**	Diagnosis, prognosis	Tissue	[171]
**HOTAIR**	Diagnosis, prognosis	Tissue, serum	[156,162,163,164,165]
**HOXA-AS2**	Prognosis	Tissue	[178]
**HOXB-AS1**	diagnosis	Tissue, serum	[167]
**HOXC13-AS**	Diagnosis, prognosis	Tissue	[168]
**MAFG-AS1**	Diagnosis, prognosis	Tissue	[173]
**PITPNA-AS1**	Prognosis	Tissue	[172]
**ROR1-AS1**	Prognosis	Tissue	[174]
**ZFAS1**	Prognosis	Tissue	[175]
**ZNF385D-AS2**	Prognosis	Tissue	[176]

**Table 3:** Antisense lncRNAs involved in chemoradioresistance.

Antisense lncRNA	Role	Drug	Molecular Mechanism	Reference
**FGD5-AS1**	Chemoresistance	Cisplatin	Sponges miR-153-39 to upregulateTWIF	[180]
**HOTAIR**	Chemoresistence	Sorafenib, cisplatin, imatinib	Sponges miR-217 to induce EMT; upregulates ABCB1; activates STAT3; sponges miR-145	[181,182,183]
**LEF1-AS1**	Chemoresistence	Cisplatin	Sponges miR-10a-5p to upregulate MSI1	[184]
**MAFG-AS1**	Chemoresistence	Sorafenib	Sponges miR-3196 to upregulate STRN4	[185]
**MKLN1-AS1**	Chemoresistence	Lenvatinib	Knockdown of MKLN1-AS increases the efficacy of lenvatinib	[92]
**TP73-AS1**	Radioresistence	Radiation	Downregulation of TP73-AS1 increases Akt, inhibiting PTEN	[186]

## Data Availability

Not applicable.
